# Shikonin derivatives cause apoptosis and cell cycle arrest in human chondrosarcoma cells via death receptors and MAPK regulation

**DOI:** 10.1186/s12885-022-09857-x

**Published:** 2022-07-12

**Authors:** Birgit Lohberger, Dietmar Glänzer, Heike Kaltenegger, Nicole Eck, Andreas Leithner, Rudolf Bauer, Nadine Kretschmer, Bibiane Steinecker-Frohnwieser

**Affiliations:** 1 Department of Orthopedics and Trauma, Medical University of Graz, 8036 Graz, Austria; 2 Ludwig Boltzmann Institute for Arthritis and Rehabilitation, Saalfelden, Austria; 3 Institute of Pharmaceutical Sciences, Department of Pharmacognosy, University of Graz, Graz, Austria

**Keywords:** Chondrosarcoma, Shikonin, Acetylshikonin, Cyclopropylshikonin, Apoptosis, Death receptors, MAPK signaling

## Abstract

**Background:**

Although chondrosarcoma is the second most common primary malignant bone tumor, treatment options are limited due to its extensive resistance to a chemo- and radiation therapy. Since shikonin has shown potent anticancer activity in various types of cancer cells, it represents a promising compound for the development of a new therapeutic approach.

**Methods:**

The dose-relationships of shikonin and its derivatives acetylshikonin and cyclopropylshikonin on two human chondrosarcoma cell lines were measured using the CellTiter-Glo®. The changes in the cell cycle were presented by flow cytometry. Protein phosphorylation and expression apoptotic markers, MAPKs and their downstream targets were analyzed using western blotting and gene expression were evaluated using RT-qPCR.

**Results:**

Chondrosarcoma cells showed a dose-dependent inhibition of cell viability after treatment with shikonin and its derivatives, with the strongest effect for shikonin and IC_50_ values of 1.3 ± 0.2 µM. Flow cytometric measurements revealed a G_2_/M arrest of the cells after treatment. Protein and gene expression analysis demonstrated a dose-dependent downregulation of survivin and XIAP, and an upregulation of Noxa, γH2AX, cleaved caspase-8, -9, -3, and -PARP. Furthermore, the expression of various death receptors was modulated. As MAPK signaling pathways play a key role in tumor biology, their phosphorylation pattern and their corresponding downstream gene regulation were analyzed. Treatment with shikonin derivatives caused an inhibition of pSTAT3 and an increase of pAKT and the MAPKs pERK, pJNK, and pp38 in a dose-dependent manner.

**Conclusions:**

These data demonstrated the significant anti-tumorigenic effect of shikonin derivatives in chondrosarcoma and encourage further research.

**Supplementary Information:**

The online version contains supplementary material available at 10.1186/s12885-022-09857-x.

## Background

Chondrosarcoma is the second most common primary malignant bone tumor after osteosarcoma and represents a heterogeneous group of locally aggressive and malignant entities. Overall survival and prognosis depend on histological grade and tumor subtype [1]. Worldwide the overall age-standardized incidence rate is 0.1–0.3 per 100,000 per year [2]. Resistance to chemo- and radiotherapy is a consequence of the underlying phenotype, which includes poor vascularization, slow division rate, and hyaline cartilage matrix that prevents access to the cells. For this reason, the therapy options are limited and complete surgical resection remains the gold standard for primary or recurrent chondrosarcoma [3, 4]. Due to the poor radiosensitivity, high doses are recommended in palliative settings, after incomplete resection or for unresectable tumors in anatomically challenging sites. Particle therapy with proton or carbon ions provide enhanced local control and patients’ survival rates [5]. However, this therapy option is only available in a few highly specialized irradiation facilities. Possible reasons for a pronounced resistance to conventional chemotherapeutic agents are the expression of multidrug resistance gene like P-glycoprotein, the high abundance of cartilaginous matrix, the expression of anti-apoptotic genes from the Bcl-2 family or the high active AKT and Src kinases [6]. From this aspect, research into novel therapeutic approaches or new substance groups is of particular importance.

Roots of *Lithospermum erythrorhizon* Siebold et Zucc or *Onosma paniculata Bur. et Franch*. are traditionally used in Chinese medicine to treat, for example, infections, and inflammatory diseases, as well as hemorrhagic diseases and contain naphthoquinone derivatives, such as shikonin and derivatives thereof. Shikonin, the most widely studied naphthoquinone derivative, has demonstrated potent anti-cancer activity in various types of cancer cells [7–11]. Known mechanisms of action are the inhibition of cell proliferation, induction of apoptosis, and reduction of cell migration and invasion potential through a variety of molecular signal transduction pathways [12, 13]. Acetylshikonin, another promising naturally occurring shikonin derivative, have also several pharmacological effects [14]. In addition, attempts have been made to optimize antitumorigenic activity by modulating the structure of naturally occurring shikonin derivatives. One of these new synthetic derivatives is cyclopropylshikonin, which has already shown promising anti-cancer activity in human melanoma cells [15].

Although there are already a number of published data on the effects of shikonin derivatives in various types of tumors, nothing is known about shikonin derivatives and the treatment of chondrosarcomas. Corresponding cellular mechanisms, the induction of apoptosis, and the regulation of mitogen-activated protein kinases (MAPKs) and signal transducer and activator of transcription 3 (STAT3) by shikonin derivatives in human chondrosarcoma cell lines have not yet been investigated. The present study addresses the effect of shikonin, and its derivatives acetylshikonin and cyclopropylshikonin, on cell viability, cell cycle distribution, apoptotic induction, death receptor expression, and the regulation of MAPK signaling pathways and their corresponding downstream targets.

## Methods

### Origin of shikonin derivatives

Acetylshikonin was isolated from dried roots of *Onosma paniculata* as described previously [12]. The plant material was acquired at the medicinal plant market in Kunming, China, and authenticated at the Kunming Institute of Botany in October 2003 and by DNA barcoding by Prof. Dr. Guenther Heubl as described previously [16]. A voucher specimen is deposited at the herbarium of the Institute for Plant Sciences, University of Graz, Austria. The collection and use of the plant material in the study was in compliance with the institutional guidelines. In brief, freshly grinded roots were extracted with petroleum ether by Soxhlet extraction. The extract was then subjected to a preparative Merck Hitachi HPLC system, consisting of a L-7100 pump, L-7200 autosampler, L-7455 diode array detector, and a D-7000 interface. Acetylshikonin was then isolated with the following column and method: VDSphere 100 RP-18 column, gradient and mobile phases: water (A) and ACN (B); 0–45 min: 70–100% B, 45–60 min: 100% B. Shikonin was purchased from Sigma Aldrich (St. Louis, MI, USA). (R)-1-(1,4-Dihydro-5,8-dihydroxy-1,4-dioxonaphthalen-2-yl)-4-methylpent-3-enyl2-cyclopropyl-2-oxoacetate (cyclopropylshikonin, CS) was prepared from shikonin as starting material as described in Kretschmer et al., 2021 [15]. In brief, acylation of shikonin was accomplished by Steglich esterification in dichloromethane with 2-cyclopropyl-2-oxoacetic and dicyclohexylcarbodiimide as coupling reagent as wells as 4-dimethylaminopyridine as catalyst. The description of substance isolation, purification, and NMR data can be found in Kretschmer et al., 2021 [15] and Lohberger et al., 2022 [17]. The purity of all compounds was measured by HPLC and/or NMR and always exceeded 95%.

### Cell culture

The human immortalized chondrosarcoma cell line SW-1353 (RRID: CVCL_0543; ATCC® HTB-94™, LGC Standards, Middlesex, UK) and Cal78 (ACC459, DSMZ, Leibniz, Germany) were cultured in Dulbecco’s-modified Eagle’s medium (DMEM-HG) supplemented with 10% FBS, 1% L-glutamine, 1% penicillin/streptomycin, and 0.25 µg amphotericin B (all GIBCO®, Invitrogen, Darmstadt, Germany). Authentication of cell lines was performed by STR profiling within the last three years. Cells were cultured in a humidified atmosphere of 5% CO_2_ at 37 °C as standard, and all experiments were performed with mycoplasma-free cells. For dose-response analysis, protein and RNA isolation the incubation period was 24 h. Since the phosphorylation process is very fast, the proteins for the analyses of the STAT3, AKT and MAPK pathways were isolated already 1 h after treatment.

### Viability assays

5 × 10^3^ chondrosarcoma cells were seeded on white 96-well plates and either used as control or treated with acetylshikonin, shikonin, or cyclopropylshikonin in various concentrations between 0.1 and 25 µM. The dose-response curves were determined using the CellTiter-Glo® Luminescence Assay (Promega, Madison, MA, USA) according the manufacturer´s protocol after a 24 h incubation period. Untreated culture media served as reference values for the background. The viability assay was performed in biological quadruplicates (*n* = 6). Absorbance values were measured with the Lumistar microplate luminometer (BMG Labtech, Ortenberg, Germany) and the corresponding IC_50_ values were calculated with SigmaPlot 14.0 (Systat Software Inc., San Jose, CA, USA) using the four-parameter logistic curve.

### Cell cycle analysis using flow cytometry

For flow cytometry analysis cells were harvested by trypsinization 24 h after treatment with acetylshikonin, shikonin, or cyclopropylshikonin and fixed with 70% ice-cold ethanol for 10 min at 4 °C. The obtained cell pellets were resuspended in propidium iodide (PI)-staining buffer (50 µl/ml PI, RNAse A) and incubated for 15 min at 37 °C. Cell cycle distribution was measured with CytoFlexLX (Beckman Coulter, Pasadena, CA, USA) and analyzed using ModFit LT software Version 4.1.7 (Verity software house). Four independent experiments were conducted in each case.

### Caspase 3/7 activity

To study the activity of caspase 3/7, chondrosarcoma cells were treated with 1.5 µM from each shikonin derivative for 1, 3, 6, 24, and 48 h and analyzed using the Caspase-Glo^®^ 3/7 Assay (Promega) according to the manufacturer´s protocol. Treatment with 1 µM staurosporine, an apoptosis inducing compound (Sigma Aldrich), was used as positive control.

### Western blot analysis

After treatment with 0.5 µM and 1.5 µM shikonin and its derivatives for 60 min for the determination of phosphorylation levels, or 0.1 to 10 µM for 24 h for the investigation of apoptotic induction and death receptors expression, whole cell protein extracts were prepared with lysis buffer (RIPA buffer, Cell Signaling Technology, Danvers, MA, USA) including a protease and phosphatase inhibitor cocktail (Sigma Aldrich). The proteins were separated by SDS-PAGE and blotted onto Amersham™ Protran™ Premium 0.45 µM nitrocellulose membrane (GE healthcare Life science, Little Chalfont, UK). Protein concentration was determined with the Pierce BCA Protein Assay Kit (Thermo Fisher Scientific) according to the manufacturer’s protocol. Primary antibodies against survivin, XIAP, Noxa, phosphorylated histone H2AX (γH2AX), cleaved-caspase-8, -9, -3, cleaved-PARP, DcR2, DcR3, FADD, TRADD, TNF-R1, TNF-R2, phospho-AKT^Ser473^, AKT, phospho-STAT3^Tyr705^, STAT3, phospho-ERK^Thr202/Tyr204^, ERK, phospho-JNK^Thr183/Tyr185^, JNK, phospho-p38^Thr180/Tyr182^, and p38 (all Cell Signaling Technology) were used over night at 4 °C. The antibody for the loading control β-actin was purchased from Santa Cruz (Santa Cruz Biotechnology, Santa Cruz, CA, USA). Blots were developed using a horseradish peroxidase- conjugated secondary antibody (Dako, Jena, Germany) at room temperature for 1 h and the Amersham™ ECL™ prime western blotting detection reagent (GE Healthcare), in accordance with the manufacturer´s protocol. Chemiluminescence signals were detected with the ChemiDocTouch Imaging System (BioRad Laboratories Inc., Hercules, CA, USA) and images were processed with the ImageLab 5.2 Software (BioRad Laboratories Inc.).

### Reverse transcription polymerase chain reaction (RT-PCR)

Total RNA was isolated 24 h after treatment with 1.5 µM shikonin or its derivatives using the RNeasy Mini Kit and DNase-I treatment according to the manufacturer’s manual (Qiagen, Hilden, Germany). Two µg RNA were reverse transcribed with the iScript-cDNA Synthesis Kit (BioRad Laboratories Inc.) using a blend of oligo(dT) and hexamer random primers. Amplification was performed with the SsoAdvanced Universal SYBR Green Supermix (Bio-Rad Laboratories Inc.) using technical triplicates and measured by the CFX96 Touch (BioRad Laboratories Inc.). The following QuantiTect primer assays (Qiagen) were used for real time RT-PCR: cdc25c, survivin, MMP2, VEGF, SOCS3, Sox9, FAK, cyclin D1, and p53. Results were analyzed using the CFX manager software for CFX Real-Time PCR Instruments (Bio-Rad Laboratories Inc., version 3.1) software and quantification cycle values (C_t_) were exported for statistical analysis. Results with Ct values greater than 32 were excluded from analysis. Relative quantification of expression levels was obtained by the ∆∆Ct method based on the geometric mean of the internal controls ribosomal protein, large, P0 (RPL) and TATA box binding protein (TBP), respectively. Expression level (C_t_) of the target gene was normalized to the reference genes (ΔC_t_), the ΔC_t_ of the test sample was normalized to the ΔC_t_ of the control (ΔΔC_t_). Finally, the expression ratio was calculated with the 2^-ΔΔCt^ method.

### Statistical analysis

Student’s unpaired t-test and the exact Wilcoxon test were used to evaluate differences between groups with the PASW statistics 18 software (IBM Corporation, Somers, NY, USA). *P*-values *p* < 0.05*, *p* < 0.01**, and *p* < 0.001*** are regarded as statistically significant.

## Results

### Effects on chondrosarcoma cell viability and cell cycle

**Fig. 1:**
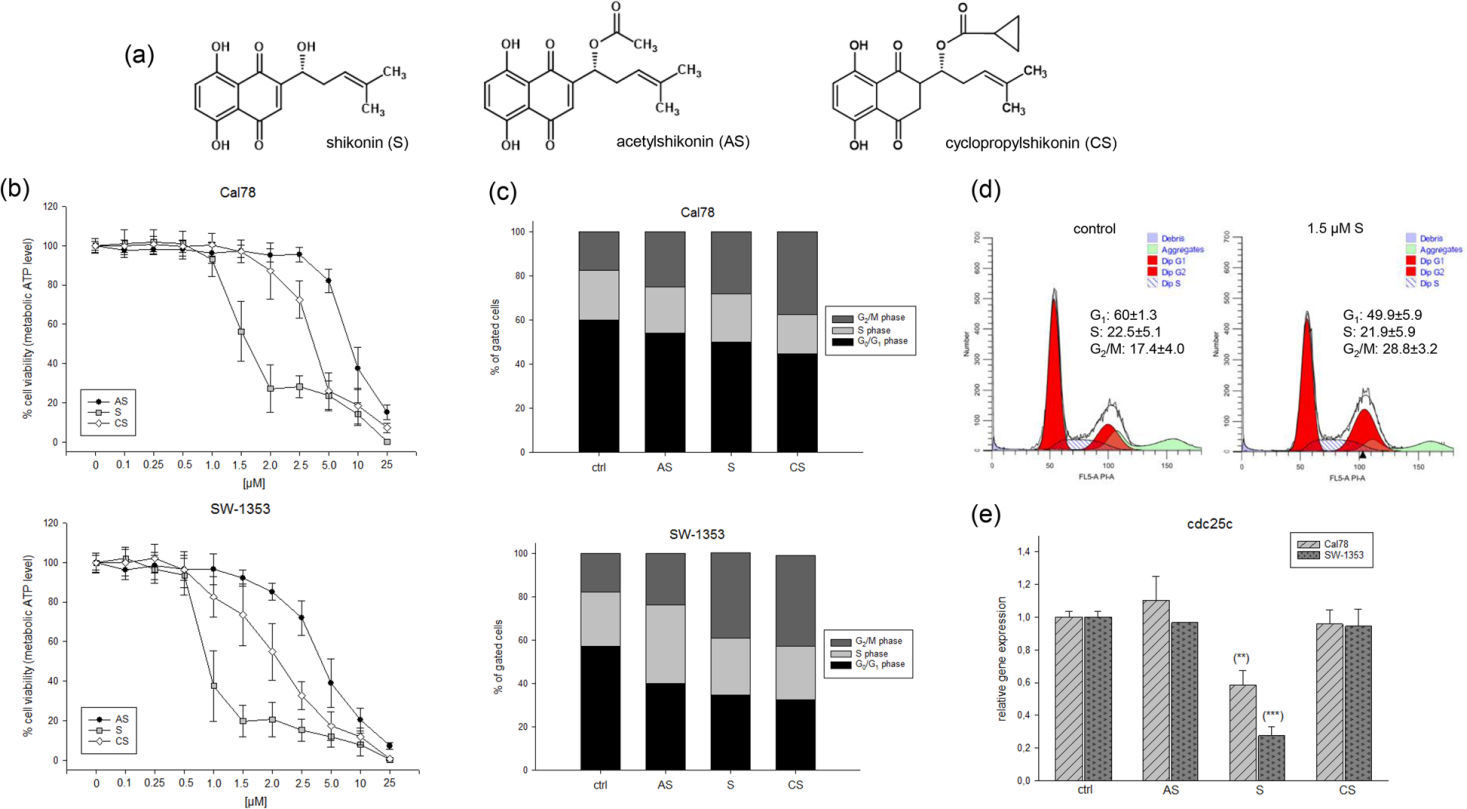
Influence of shikonin derivatives on chondrosarcoma viability and cell cycle distribution. **a** Chemical structures of shikonin (S), acetylshikonin (AS), and cyclopropylshikonin (CS); **b** Cell growth of two chondrosarcoma cell lines was inhibited in a dose-dependent manner by shikonin derivatives (mean ± SD, *n* = 6, measured in biological quadruplicates). **c** The statistical evaluation of cell cycle distribution after treatment with the IC_50_ concentrations of shikonin derivatives is shown in stacked bar charts. Cell populations in G_0_/G_1_, S, and G_2_/M phases are given as percentage of total cells (mean of *n* = 3). Treatment caused a dose dependent significant decrease in the number of cells in G_0_/G_1_ phase (black) and S phase (light grey), which was accompanied by a pronounced increase of cells in G_2_/M phase (dark grey). **d** Representative flow cytometry cell cycle measurements 24 h after treatment with shikonin. **e** Relative gene expression of the cell cycle regulator cdc25c after treatment with shikonin for 24 h revealed a highly significant reduction in SW-1353 (light grey striped) and Cal78 (dark grey dotted) cells (mean ± SD, *n* = 6, measured in triplicates). Statistical significances are defined as follows: * *p* < 0.05; ** *p* < 0.01; *** *p* < 0.001

To study the effects of shikonin and its derivatives (Fig. 1a), chondrosarcoma cells were treated with various concentrations of the compounds in focus and the dose-response relationship was analyzed. Both cell lines showed a dose-dependent inhibition of cell viability after treatment with shikonin derivatives (Fig. 1b). The strongest effects after 24 h were found for shikonin (IC_50_ 1.5 µM for Cal78 and 1.1 µM for SW-1353). The IC_50_ values of acetylshikonin were 3.8 µM and 1.5 µM and for the novel derivative cyclopropylshikonin 2.9 µM and 1.2 µM, respectively. Flow cytometric measurements revealed a G_2_/M arrest of the cells after treatment with the calculated IC_50_ concentrations of all three derivatives, whereby shikonin and cyclopropylshikonin showed a stronger effect than acetylshikonin (Fig. 1c). A representative measurement with the corresponding percentages of cells in the G_0_/G_1_, S and G_2_/M phase is shown in Fig. 1d. The G_2_/M arrest of cells induced via treatment with shikonin can be attributed to a reduction in cdc25c expression (Fig. 1e).

### Effects on apoptotic induction by investigating survivin, XIAP, Noxa, and the DNA damage marker γH2AX

**Fig. 2:**
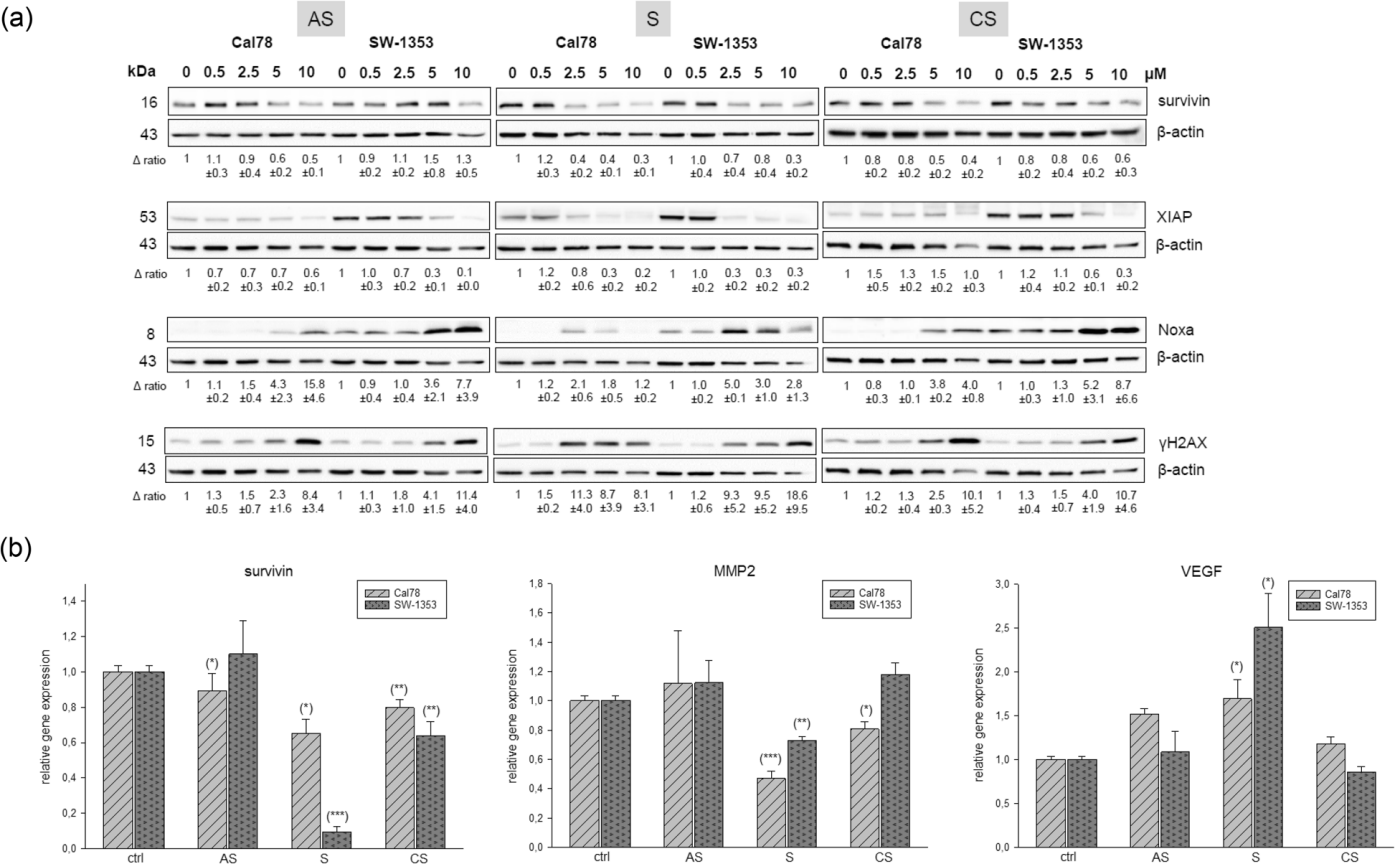
Induction of apoptotic key players. **a** Protein expression of survivin, XIAP, Noxa, and the DNA damage marker γH2AX. The apoptotic key players were evaluated by immunoblotting under control conditions (0) and after treatment with 0.5–10 µM acetylshikonin (AS), shikonin (S), and cyclopropylshikonin (CS). β-actin was used as loading control. Δ ratio, fold change normalized to non-treated controls (mean ± SD of *n* = 3). Full-length blots are presented in Supplementary Fig. S1. **b** Relative gene expression of survivin, the metastasis marker MMP2, and angiogenic marker VEGF after treatment with shikonin derivatives for 24 h in SW-1353 (light grey striped) and Cal78 (dark grey dotted) cells (mean ± SD, *n* = 6, measured in triplicates). Statistical significances are defined as follows: * *p* < 0.05; ** *p* < 0.01; *** *p* < 0.001

To investigate the induction of apoptosis in chondrosarcoma cells, whole cell lysates for western blot analysis were extracted 24 h after treatment of cells with 0.5 to 10 µM shikonin derivatives. Fold changes normalized to untreated controls (Δ ratio; mean ± SD of *n* = 3) were presented. Shikonin derivatives dose-dependently downregulated the protein expression of survivin and the X-linked inhibitor of apoptosis (XIAP), whereas the pro-apoptotic gene Noxa and the DNA damage marker γH2AX were upregulated (Fig. 2a). Although the progression of both cell lines is basically similar, minor differences in sensitivity can be observed. Confirming the protein analyses, a highly significant reduction in survivin gene expression was observed in both chondrosarcoma cell lines after treatment with 1.5 µM of the shikonin derivatives (Fig. 2b). Untreated cells were used as control (ratio = 1) (mean ± SEM; *n* = 6). Gene expression analysis revealed a significant downregulation of the metastatic marker MMP2 after treatment with 1.5 µM shikonin or cyclopropylshikonin for 24 h. Treatment with shikonin, on the other hand, upregulated the angiogenesis marker VEGF (Fig. 2b).

### Caspase activity and PARP cleavage as hallmark of apoptosis

**Fig. 3:**
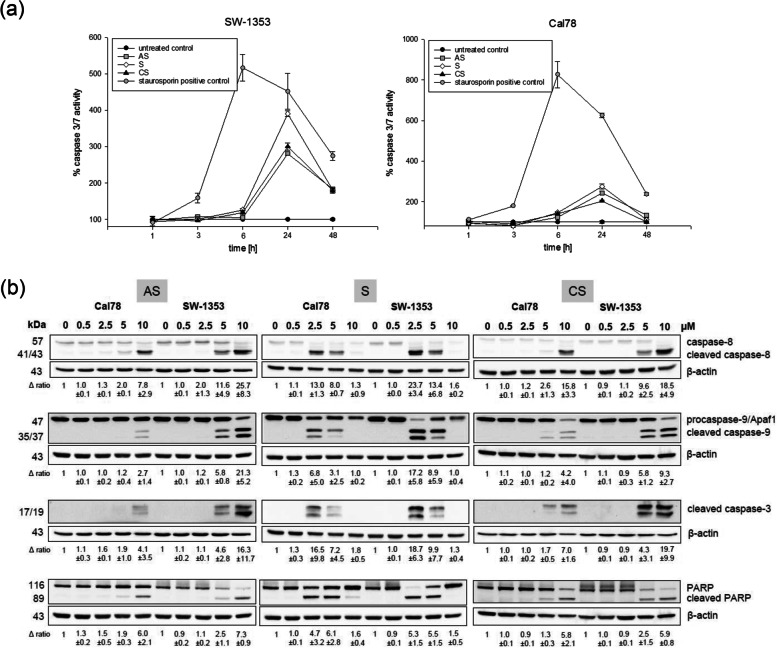
Activity of the caspases. **a** Caspase 3/7 activity was measured after 1–48 h. In both chondrosarcoma cell lines, caspase 3/7 activity level peaked after 24 h (Caspase-Glo^®^ assay, mean ± SD, *n* = 2, measured in biological quadruplicates). **b** Western blot analyses were used to verify cleaved caspase-3, -8, and − 9 expression, respectively PARP cleavage at the protein level. One representative blot out of three is shown and β-actin was used as loading control. ∆ represents fold change normalized to controls (mean ± SD; *n* = 3). Higher concentrations of the shikonin derivatives induced caspase-8 and-9 activity and the cleavage of caspase 3 and PARP. All full-length blots are presented in Supplementary Fig. S1

Caspases 3 and 7 are activated as central players during apoptosis. To determine the best time frame for a possible apoptotic induction, the caspase 3/7 Glo activity assay was performed. In both chondrosarcoma cell lines, caspase 3/7 activity peaked after 24 h (mean ± SD; *n* = 2; measured in quadruplicates) (Fig. 3a). For this reason, this time point was used for all further apoptosis analyses. Staurosporine was used as positive control and showed a rapid and strong increase in caspase 3/7 activity. After treatment with 0.5, 2.5, 5, or 10 µM for 24 h protein analyses revealed increasing cleavage of caspase-8, caspase-9, caspase 3, and PARP at higher concentrations (5 and 10 µM) of all derivatives. Shikonin showed this effect already at a concentration of 2.5 µM. One representative blot out of three is shown in Fig. 3b and β-actin was used as loading control. “∆ ratio” represents fold change normalized to controls (mean ± SD; *n* = 3).

### Shikonin derivatives affected the expression of death receptors

**Fig. 4:**
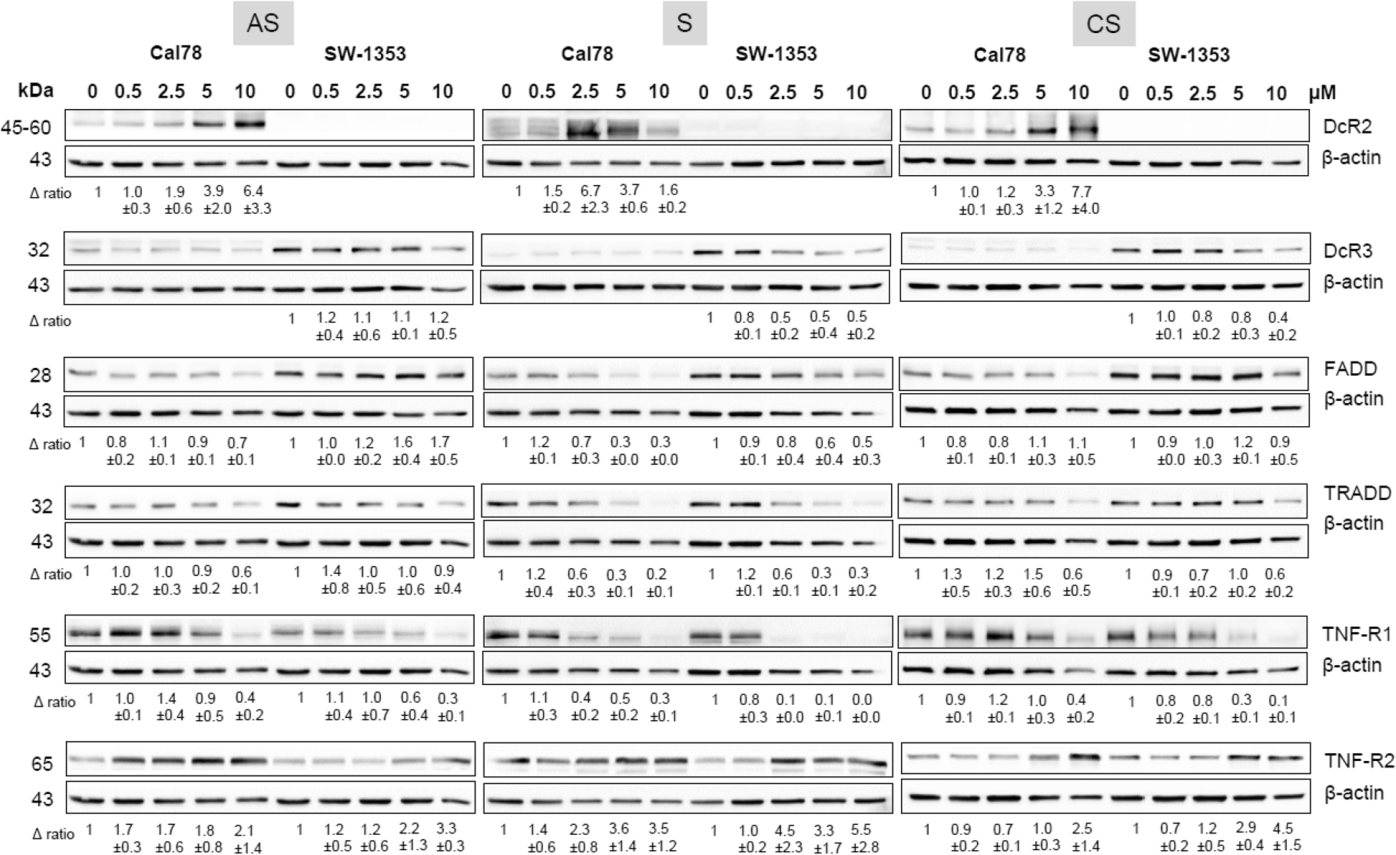
Influence on protein expression of death receptors. Relative protein expression analysis of DcR2, DcR3, FADD, TRADD, TNF-R1, and TNF-R2 after treatment with 0.5 µM and 1.5 µM acetylshikonin (AS), shikonin (S), and cyclopropylshikonin (CS) for 24 h in chondrosarcoma cells. Untreated control cells (ctrl) served as reference value and β-actin as loading control (mean ± SD, *n* = 3). All full-length blots are presented in Supplementary Fig. S1

Death receptor protein expression was analyzed using western blotting. It could be shown that not all death receptors are expressed by both cell lines (Fig. 4). Treatment of chondrosarcoma cells with increasing concentrations of shikonin derivatives (0.5, 2.5, 5, or 10 µM) for 24 h resulted in an increase of DcR2 and TNF-R2 expression. In contrast, the expression of DcR3 and TNF-R1 showed decreasing trends and for FADD and TRADD, a change was detected only for the most potent shikonin. One representative blot out of three is shown in Fig. 4 and β-actin was used as loading control. “∆ ratio” represents fold change normalized to controls (mean ± SD; *n* = 3).

### MAPK regulation by shikonin derivatives

**Fig. 5:**
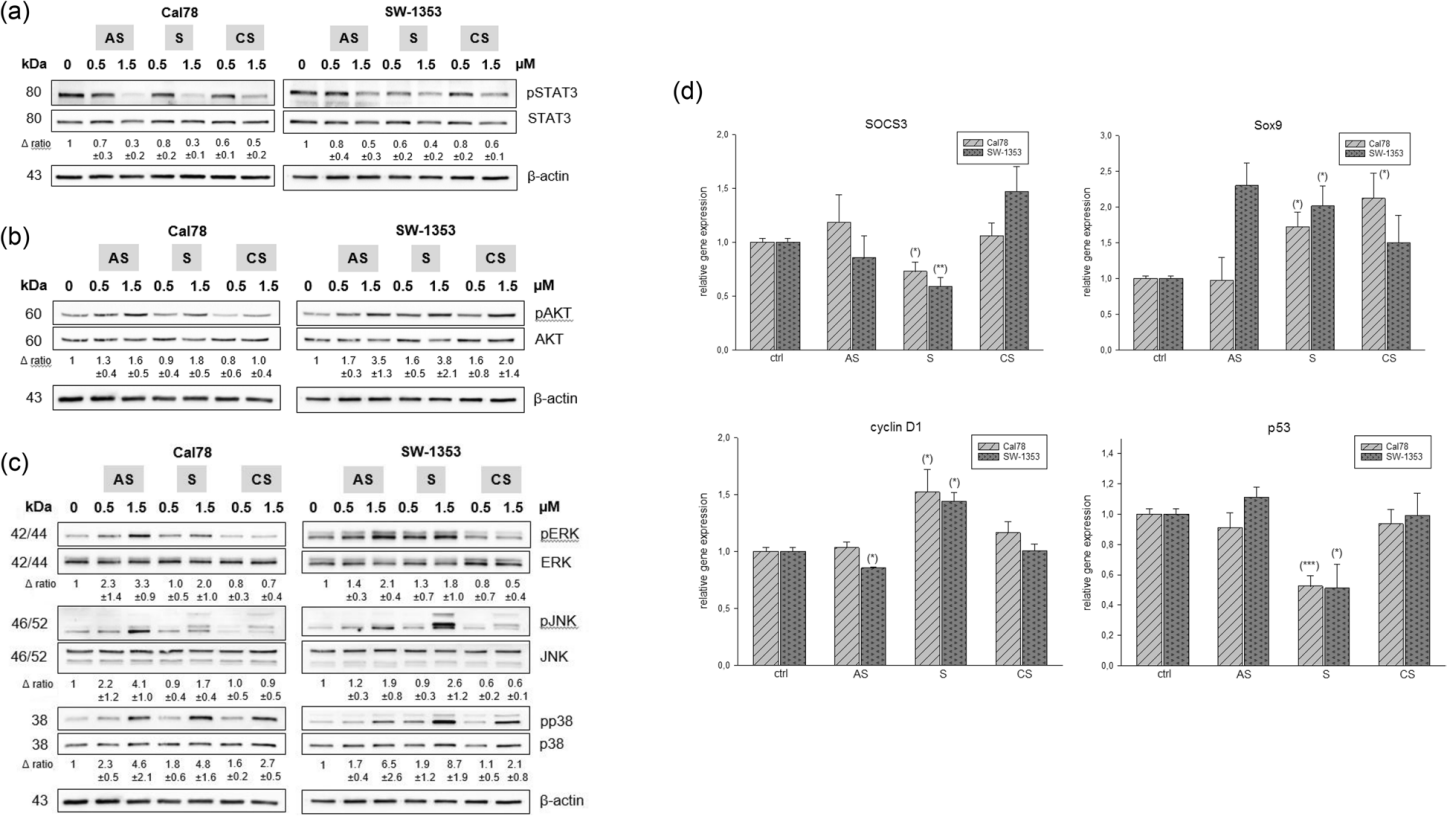
Regulation of protein phosphorylation of STAT3, AKT, the MAPKs ERK/JNK/p38 and their influence on downstream targets. **a** The STAT3, **b** AKT, **c** ERK/JNK/p38 protein phosphorylation was evaluated by immunoblotting under control conditions (ctrl) and in the presence of 0.5 µM and 1.5 µM acetylshikonin (AS), shikonin (S), and cyclopropylshikonin (CS) for 1 h in SW-1353 and Cal78 cells. Δ, represents the ratio of phosphorylated to unphosphorylated proteins (mean ± SD, *n* = 3). β-actin was additionally used as loading control. **d** Relative gene expression of the downstream targets cyclin D1, cMyc, SOCS3, and Sox9 after treatment with shikonin derivatives for 24 h in SW-1353 (light grey striped) and Cal78 (dark grey dotted) cells (mean ± SD, *n* = 6, measured in triplicates). Statistical significances are defined as follows: * *p* < 0.05; ** *p* < 0.01; *** *p* < 0.001

To investigate the ability of shikonin derivatives to affect MAPK phosphorylation levels, whole cell lysates of chondrosarcoma cells were extracted 1 h after treatment with 0.5 and 1.5 µM and prepared for western blot analysis. With increasing concentrations of shikonin derivatives, a dose-dependent inhibition of STAT3 phosphorylation was observed at protein level (Fig. 5a). In contrast, the phosphorylation of the serine/threonine kinase AKT is increased especially at the higher concentrations (Fig. 5b). To determine the underlying mechanism of apoptosis induction, protein analysis was performed for ERK, JNK, and p38 proteins, which are major participants in the MAPK pathway. An increased phosphorylation of pERK, pJNK, and pp38 was observed in shikonin derivatives treated cells compared with untreated controls (Fig. 5c). One representative blot out of three is shown and β-actin was used as loading control. ∆ represents the ratio of phosphorylated to unphosphorylated MAPKs (mean ± SD; *n* = 3). The gene expression analysis of the STAT 3 downstream targets SOCS3, Sox9, cyclin D1, and p53 were presented in Fig. 5d. After treatment with shikonin, both chondrosarcoma cell lines revealed a significant reduction in SOCS3 expression. The two derivatives acetylshikonin and cyclopropylshikonin showed no significant differences. Sox9 and cyclin D1 were significantly upregulated, again mainly by shikonin treatment for 24 h. For gene expression analysis untreated cells were used as control (ratio = 1) (mean ± SEM; *n* = 6).

## Discussion

As chondrosarcomas largely resist conventional chemo- and radiotherapy, the investigation of new substance groups and their underlying cellular mechanisms is of utmost importance. Roots of *Lithospermum erythrorhizon*, which are used in traditional Chinese medicine, have been reported to show pronounced anti-cancer effects. Shikonin, one of the main active ingredients, is a highly interesting target molecule with a broad application prospect and a realistic potential of clinical use [10]. The cytotoxic effects of shikonin derivatives on human chondrosarcoma cells were accessed by CellTiter-Glo® assay, which determines the number of metabolically active cells via ATP quantification. Both cell lines showed a dose-dependent inhibition of cell viability and IC_50_ values, which are lower than those determined for melanoma cells and human embryonic kidney cells [15]. This suggests that human chondrosarcoma cells might be more sensitive to shikonin than other tumor entities. Flow cytometric measurements revealed an arrest of chondrosarcoma cells in the G_2_/M phase of the cell cycle. Cell cycle checkpoints help ensure the accuracy of DNA replication and allow progression through the cell cycle or arrest to allow time for DNA repair [18]. Treatment with shikonin inhibited the expression of cdc25c, which caused the G_2_/M checkpoint proteins cdc2/cyclin B1 to remain in an inactive phosphorylated state. These observations are consistent with those of Zhang et al., 2019, who demonstrated in different tumor entities that shikonin induces cell cycle arrest mediated by cdc25 inhibition [19].

Apoptotic induction, inhibition of migration properties, and regulation of MAPK phosphorylation are further important cellular mechanisms in defining the anti-cancer activity of shikonin [10]. We could show that shikonin derivatives induced apoptosis with increasing concentrations between 0.5 and 10 µM in chondrosarcoma cells. Activation of caspases triggers programmed cell death, resulting in cell membrane swelling, cell contraction, chromatin condensation, and DNA degradation [20]. In addition to caspase activation, key factors such as surviving (BIRC5) or XIAP are considered to be features of progressive apoptosis. Survivin, as a member of the apoptosis inhibitor family, inhibits caspase activation and leads to negative regulation of apoptosis and is therefore essential for chondrosarcoma cell survival [21]. Survivin is highly expressed in high grade chondrosarcomas [22]. As the concentrations are largely consistent with the calculated IC_50_ values, the highly significant downregulation of survivin by shikonin derivatives might be a potential mechanism for inducing apoptosis. This result was demonstrated at both the gene expression and protein expression levels. Moreover, shikonin derivatives dose-dependently regulated the expression of X-linked apoptosis inhibitor (XIAP) in chondrosarcoma cells, leading to damage of mitochondrial membrane potential and subsequent induction of apoptosis. Wang et al. were able to demonstrate a similar effect in non-small-cell lung cancer cells [23]. As we have shown in a previous work in melanoma cells, increased concentrations of shikonin derivatives led to a dose-dependent increase in Noxa expression in our cell system [24]. Cell death induced by shikonin derivatives can also be triggered by the induction of DNA double-strand breaks (DSB), as indicated by elevated levels of the phosphorylated histone variant γH2AX, a biomarker for DSB [25]. Studying the protein phosphorylation of this DNA damage biomarker, a concentration dependent increase in the expression was detected for both chondrosarcoma cell lines under the treatment of 2.5 to 10 µM of shikonin derivatives. Matrix metalloproteinases (MMPs) are actively involved in the overall metastasis process due to their biological functions. These include their ability to degrade extracellular matrix components and to interact with growth factors such as cytokines and chemokines [26]. Shikonin and cyclopropylshikonin decreased significantly the gene expression of MMP2 (Gelatinase-A). Angiogenesis also plays an important role in tumor growth and metastasis of a variety of malignancies. One of the best characterized angiogenesis factors are endothelial growth factors, such as “vascular endothelial growth factor” (VEGF) [27]. After shikonin treatment, a significant increase in VEGF expression was observed in both chondrosarcoma cell lines.

Successful eradication of cancer cells by nonsurgical means is ultimately approached via induction of apoptosis. The family of caspases is activated in the early stages of apoptosis and cleaves key cellular components required for normal cell function. These include structural proteins of the cytoskeleton and nuclear proteins such as DNA repair enzymes [28]. After treatment with the shikonin derivatives, the results of the caspase 3/7 activity assay showed a significant spike after only 24 h. For this reason, this point in time was used for all further analyses. Staurosporine, a protein kinase inhibitor, was used as positive control and showed a rapid and strong increase in caspase 3/7 activity. This compound has been characterized as a strong inducer of apoptosis in many different cell types [29]. While caspase-9 activation constitutes the intrinsic pathway, the extrinsic pathway requires caspase-8 activation. Our data revealed that the activity of caspase-8 and caspase-3 was significantly increased in a concentration-dependent fashion in chondrosarcoma cells, indicating the apoptosis was induced by shikonin derivatives in our cell system by the extrinsic pathway. During the apoptosis process caspase-3 and − 7 are executioner caspases and initiate the PARP cleavage, whereby cleavage of PARP is considered a hallmark of apoptosis. In our cell system the cleavage of PARP could also be observed. In colorectal cancer cells, similar effects could be shown after treatment with 1.5 µM shikonin also after 24 h [30], likewise in cholangiocarcinoma cells after treatment with 5 µM shikonin [31].

Death receptors have been identified as a subgroup of the TNF-receptor superfamily with a predominant function in induction of apoptosis. The receptors are characterized by an intracellular region, called the death domain, which is required for the transmission of the cytotoxic signal. These are linked to the caspases in many ways within the apoptosis pathway [32]. TNF-receptors (TNF-R) promote apoptosis via the adaptor proteins TRADD/FADD and the activation of caspase-8. Chondrosarcoma cells showed an increase in DcR2 and TNF-R2 expression after treatment with shikonin derivatives, although not both cell lines expressed all death receptors. DcR3 and TNF-R1 expression, on the other hand, showed decreasing trends, and FADD and TRADD revealed a change only for the most potent shikonin. This is consistent with results observed in lung cancer cell lines by Han et al. [33].

The mitogen-activated protein kinases (MAPKs) are a family of serine/threonine kinases that transduce signals from the cell membrane to the nucleus. Depending on cell type, stimuli and the latency of activation, MAPK signalling may either protect or enhance sensitivity to apoptosis, differentiation and proliferation [34]. Lee et al. reported a MAPK pathway-mediated induction of apoptosis of melanoma cells after shikonin treatment [35]. With this background, western blot analyses were performed to determine whether shikonin-induced apoptosis in chondrosarcoma cells is mediated by MAPK signaling pathways. The inhibitory effect of STAT3 phosphorylation by shikonin has already been shown in various tumor types, such as pancreatic cancer [36], melanoma [37], or lung cancer [38]. We were able to demonstrate, especially in Cal78 chondrosarcoma cells, a significant reduction of STAT3 phosphorylation after treatment with all shikonin derivatives. As phosphorylation is a very fast effect, the 1 h time point was chosen. In contrast, the phosphorylation level of the serine/threonine kinase AKT is increased. It has already been shown, that the AKT pathway play an important role in shikonin-induced apoptosis in several types of cancer. Cleavage of AKT occurs during apoptosis [39] suggests that either a level of baseline AKT signaling is vital for cell survival or that AKT activation occurs during apoptosis and acts as a “brake” on the process. Fast activation of AKT mediates survival of cells in various death settings and executed primarily through the mitochondrial apoptotic pathway. According to enzyme kinetics, the characteristic phosphorylation time of Ser-473 and Thr-308 at AKT kinase is 20–150 s. For this reason, we see an activation of AKT phosphorylation after 1 h treatment with shikonin derivatives.

In chondrosarcoma cells increased phosphorylation was also detected in the MAPKs ERK, JNK, and p38. This type of regulation by shikonin has been previously described for human NB4 leukemia cells, where it resulted in increased phosphorylation of JNK and p38 [40]. The suppressor of cytokine signaling 3 (SOCS3) is a negative regulator of the JAK/STAT signaling pathway and was significantly increased by shikonin treatment. Whereas, the other two derivatives showed no significant differences. Decreased STAT3 phosphorylation upregulated Sox9 expression as it physically interacts with the promoter in response to stimulation. Cyclin D1 is an important regulator of the cell cycle as it promotes progression through G1-S phase. In human lung cancer and pancreatic cells shikonin suppressed cell proliferation through modulating the expression of cell cycle regulators like cyclin D1 or cMyc [36, 41]. Although apoptosis induction through ERK activation has not been clearly elucidated, up-regulation of p53 by activated ERK during apoptosis has been demonstrated to promote apoptosis, depending on the cell line and stimuli [42].

## Conclusions

Our results demonstrate for the first time that shikonin and its derivatives acetylshikonin and cyclopropylshikonin have extensive anti-tumor efficacy in chondrosarcoma and affect cell viability, cell cycle distribution, and apoptotic induction via caspase cleavage and death receptors regulation. Furthermore, the phosphorylation level of STAT3 was inhibited, whereas pAKT and the MAPKs pERK, pJNK, and pp38 were increased. These extensive cellular changes provide a valuable basis for further research.

## Supplementary Information

**Additionl file 1.**

## Data Availability

The datasets supporting the conclusions of this article are included within the article (and its additional file).
