# Supplementary material for: Shikonin derivatives cause apoptosis and cell cycle arrest in human chondrosarcoma cells via death receptors and MAPK regulation
Source: BMC Cancer. 2022 Jul 12;22:758. doi: 10.1186/s12885-022-09857-x (PMC9275282; doi:10.1186/s12885-022-09857-x)
Supplement: Supplementary file 1 — Additionl file 1. [file 12885_2022_9857_MOESM1_ESM.pdf]

Supplementary data uncropped blots

Lohberger et al.

Figure2a

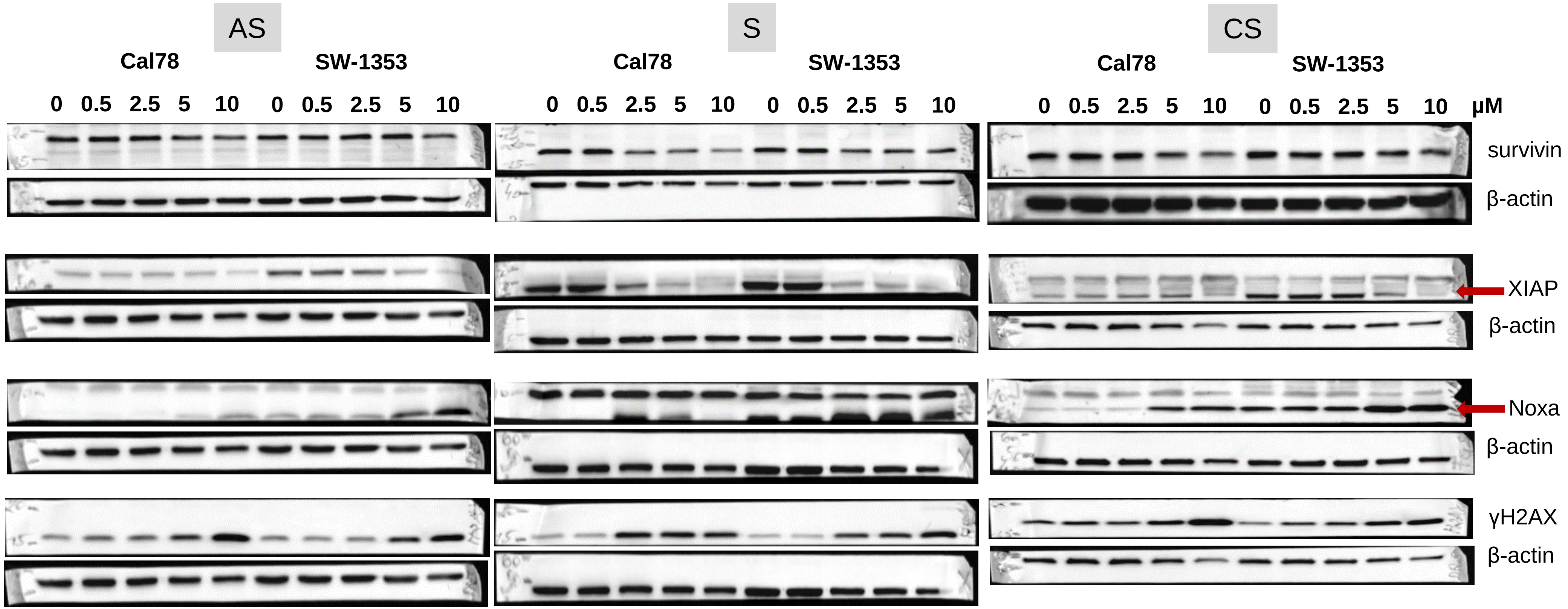
AS
S
CS
Cal78
SW-1353
Cal78
SW-1353
Cal78
SW-1353
0 0.5 2.5 5 10 0 0.5 2.5 5 10
0 0.5 2.5 5 10 0 0.5 2.5 5 10
0 0.5 2.5 5 10 0 0.5 2.5 5 10 μM
survivin
β-actin
XIAP
β-actin
Noxa
β-actin
γH2AX
β-actin

Figure3b_part 1

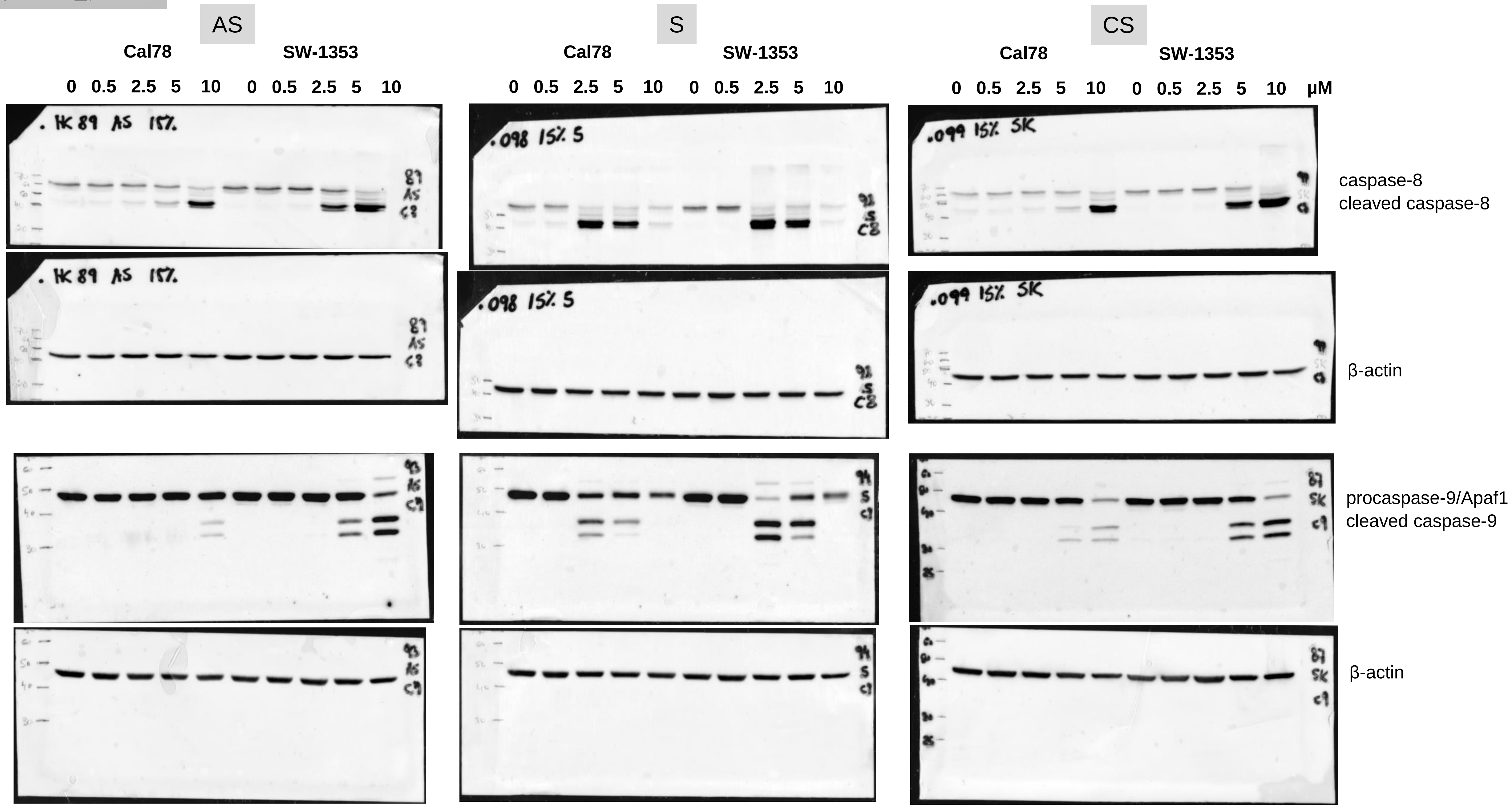

Figure3b_part 2

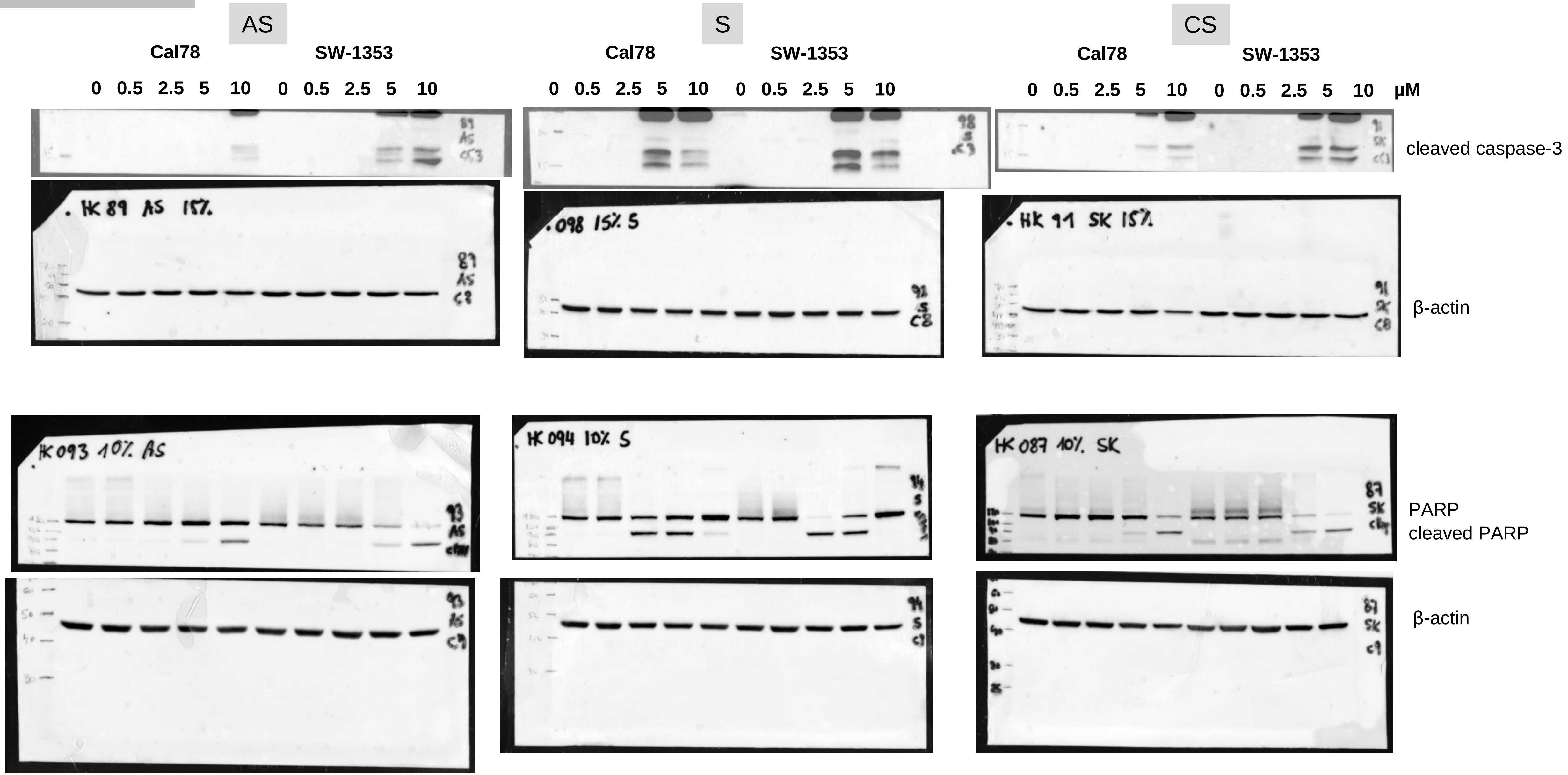

Figure 4

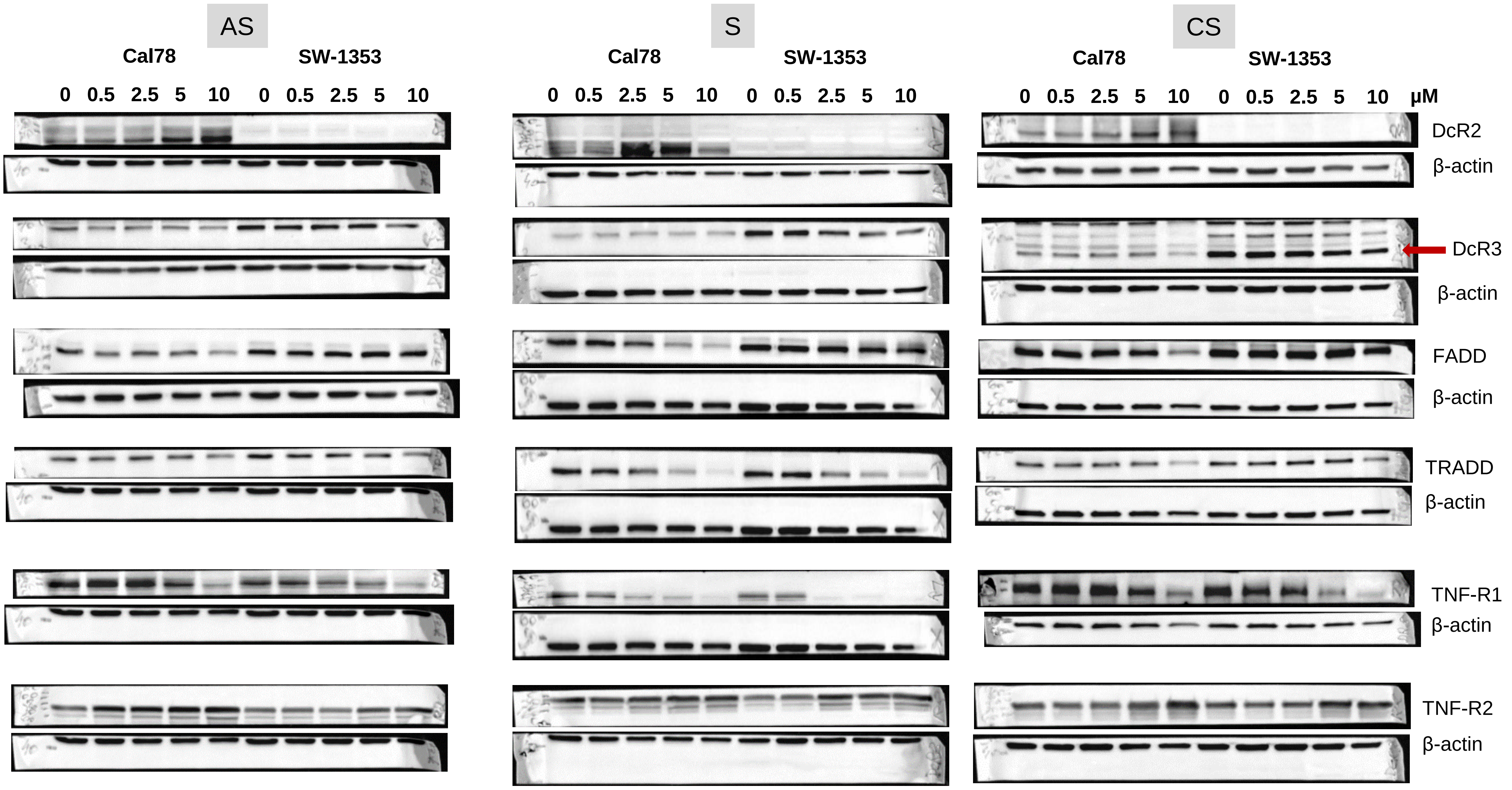
AS
S
CS
Cal78
SW-1353
0 0.5 2.5 5 10 0 0.5 2.5 5 10 μM
DcR2
β-actin
DcR3
β-actin
FADD
β-actin
TRADD
β-actin
TNF-R1
β-actin
TNF-R2
β-actin

Figure 5a

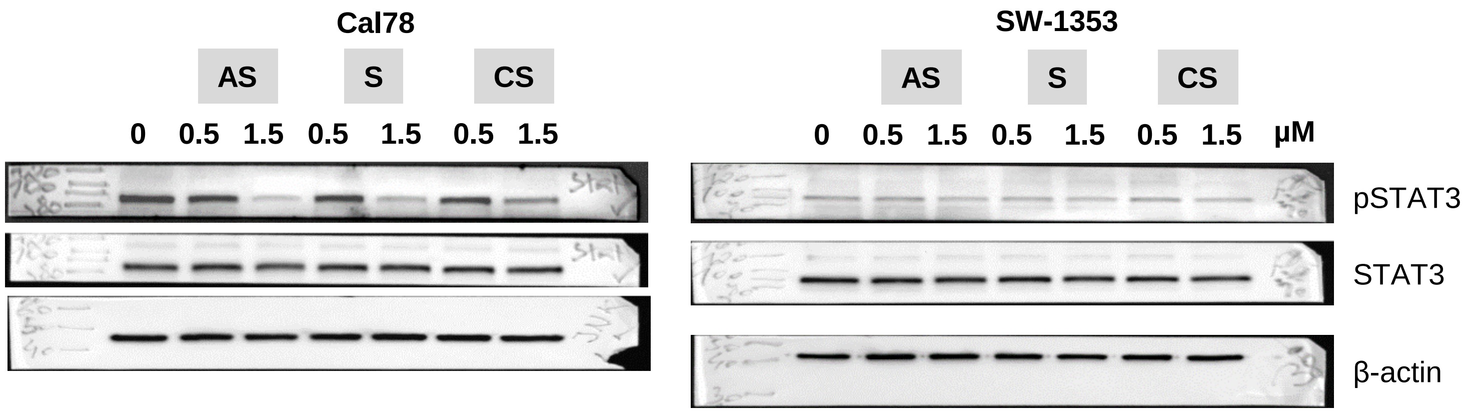

Figure 5b

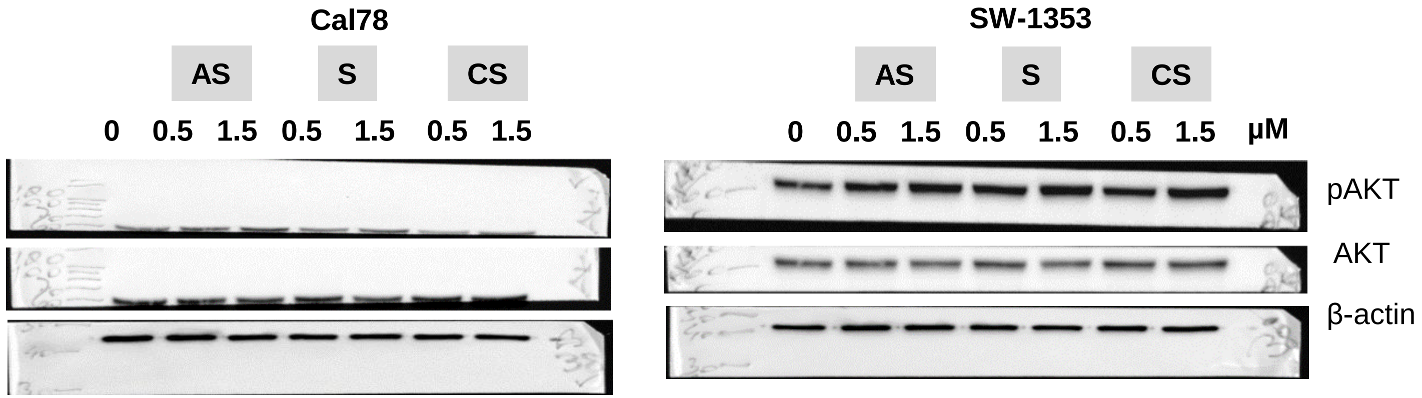

Figure 5c

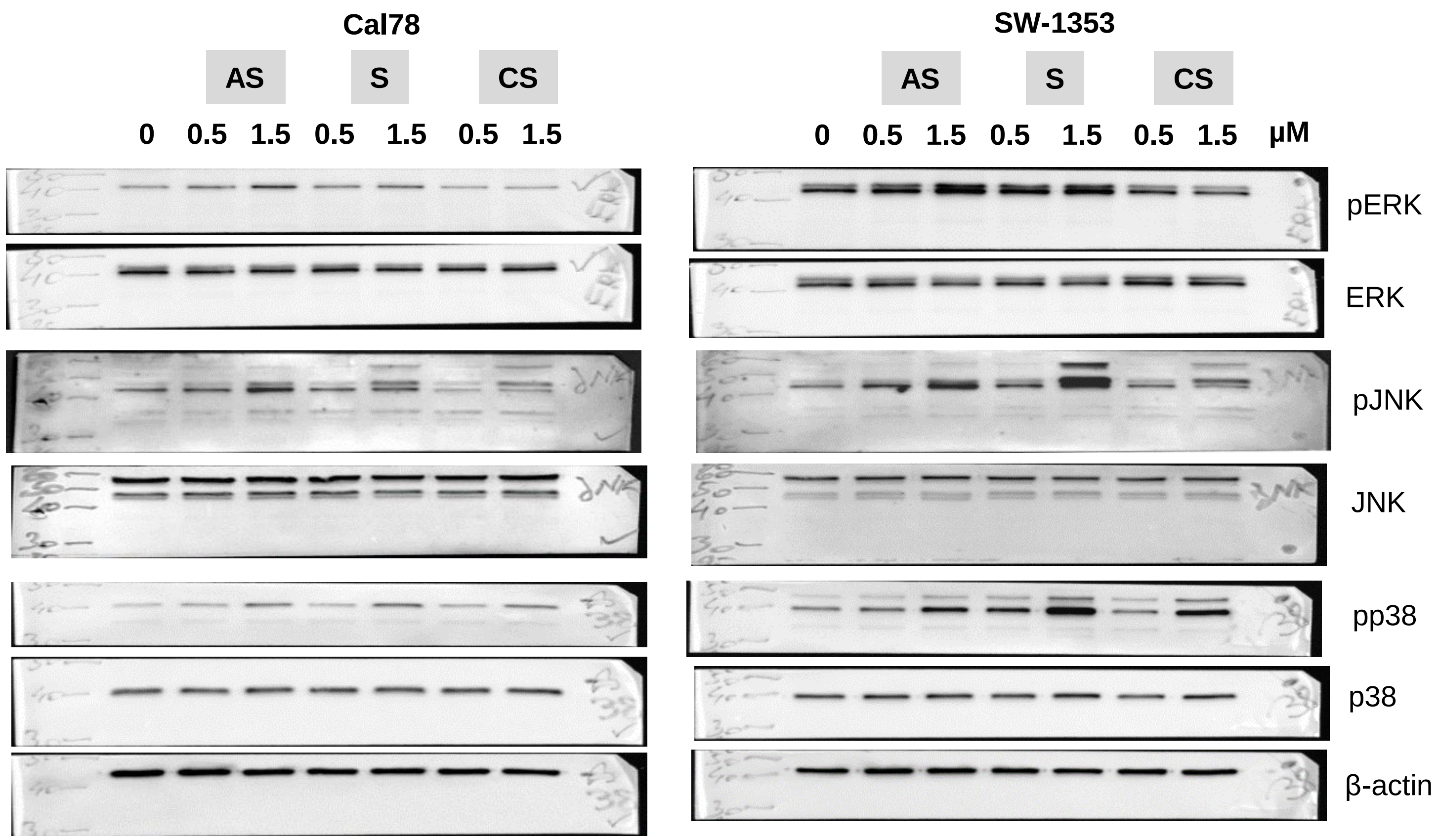
Cal78
AS
S
CS
0
0.5
1.5
0.5
1.5
0.5
1.5
SW-1353
AS
S
CS
0
0.5
1.5
0.5
1.5
0.5
1.5
μM
pERK
ERK
pJNK
JNK
pp38
p38
β-actin
